# Pleiotropic Effects of Atorvastatin Result in a Downregulation of the Carboxypeptidase U System (CPU, TAFIa, CPB2) in a Mouse Model of Advanced Atherosclerosis

**DOI:** 10.3390/pharmaceutics13101731

**Published:** 2021-10-19

**Authors:** Karen Claesen, Lynn Roth, Joachim C. Mertens, Karlijn Hermans, Yani Sim, Dirk Hendriks

**Affiliations:** 1 Laboratory of Medical Biochemistry, Department of Pharmaceutical Sciences, University of Antwerp, 2610 Wilrijk, Belgium; karen.claesen@uantwerpen.be (K.C.); mertens-joachim@gmail.com (J.C.M.); karlijn.hermans@telenet.be (K.H.); yani.sim@uantwerpen.be (Y.S.); 2 Laboratory of Physiopharmacology, Department of Pharmaceutical Sciences, University of Antwerp, 2610 Wilrijk, Belgium; lynn.roth@uantwerpen.be

**Keywords:** thrombin activatable fibrinolysis inhibitor, carboxypeptidase B2, HMG-CoA reductase inhibitors, animal models, carboxypeptidase U, fibrinolysis

## Abstract

Statins (hydroxymethyl-glutaryl-CoA-reductase inhibitors) lower procarboxypeptidase U (proCPU, TAFI, proCPB2). However, it is challenging to prove whether this is a lipid or non-lipid-related pleiotropic effect, since statin treatment decreases cholesterol levels in humans. In apolipoprotein E-deficient mice with a heterozygous mutation in the fibrillin-1 gene (ApoE^−/−^Fbn1^C1039G+/−^), a model of advanced atherosclerosis, statins do not lower cholesterol. Consequently, studying cholesterol-independent effects of statins can be achieved more straightforwardly in these mice. Female ApoE ^−/−^Fbn1C^1039G+/−^ mice were fed a Western diet (WD). At week 10 of WD, mice were divided into a WD group (receiving WD only) and a WD + atorvastatin group (receiving 10 mg/kg/day atorvastatin +WD) group. After 15 weeks, blood was collected from the retro-orbital plexus, and the mice were sacrificed. Total plasma cholesterol and C-reactive protein (CRP) were measured with commercially available kits. Plasma proCPU levels were determined with an activity-based assay. Total plasma cholesterol levels were not significantly different between both groups, while proCPU levels were significantly lower in the WD + atorvastatin group. Interestingly proCPU levels correlated with CRP and circulating monocytes. In conclusion, our results confirm that atorvastatin downregulates proCPU levels in ApoE^−/−^Fbn1^C1039G+/−^ mice on a WD, and evidence was provided that this downregulation is a pleiotropic effect of atorvastatin treatment.

## 1. Introduction

The zymogen procarboxypeptidase U (proCPU, TAFI, proCPB2) circulates in plasma and is converted into the active enzyme carboxypeptidase U (CPU, TAFIa, CPB2) by thrombin(-thrombomodulin) or plasmin [1,2]. CPU potently attenuates fibrinolysis through the cleavage of C-terminal lysines on partially degraded fibrin, thereby interfering with efficient plasminogen activation [2]. Lowering proCPU levels will improve the fibrinolytic capacity and is therefore expected to be beneficial in individuals at high risk for thromboembolic diseases. Furthermore, CPU has been shown to modulate inflammation through cleavage of proinflammatory mediators (e.g., C3a, C5a, bradykinin) [3,4].

Statins (inhibitors of 3-hydroxy-3-methyl-glutaryl coenzyme A reductase) exert cardiovascular protective effects that are independent of the lowering of LDL-cholesterol, including profibrinolytic effects such as the reduction of proCPU levels in hypercholesterolemic patients [5,6,7,8]. However, it is challenging to prove whether this is a lipid or a non-lipid-related pleiotropic effect, since lowering cholesterol is inherent to statin treatment in humans. In apolipoprotein E-deficient mice with a heterozygous mutation in the fibrillin-1 gene (ApoE^−/−^Fbn1^C1039G+/−^) on a Western diet (WD)—a model of advanced atherosclerosis—statins do not significantly lower cholesterol [9,10]. Therefore, this model was used in the current study to investigate the effect of atorvastatin treatment on proCPU biology in a cholesterol-independent setting.

## 2. Materials and Methods

This research is a post-hoc analysis within the study of Roth et al. on the cholesterol-independent effects of atorvastatin on the prevention of cardiovascular morbidity and mortality in a mouse model of atherosclerotic plaque rupture [10].

### 2.1. Animals and Study Protocol

Female ApoE^−/−^ Fbn1^C1039G+/−^ mice were housed in a temperature-controlled room with a 12 h light/dark cycle and had free access to water and food. At an age of 6 weeks, all mice were fed a Western diet (WD; 4021.90, AB Diets, Woerden, the Netherlands) for 10 weeks; then, the ApoE^−/−^ Fbn1^C1039G+/−^ mice were randomly divided into two groups. A WD was continued in both groups for another 15 weeks, but only in one group was the diet supplemented with atorvastatin (10 mg/kg/day, Pfizer, New York, NY, USA). Groups are referred to as mice on WD (N = 21) and atorvastatin-treated mice (N = 20), respectively.

On the 25th week from the initiation of WD, mice were anesthetized by an intraperitoneal injection of ketamine (100 mg/kg) and xylazine (10 mg/kg) for blood sampling. Blood was collected via the retro-orbital plexus into tubes containing ethylenediaminetetraacetic acid (EDTA; final concentration 5 mM) and kept on ice. Afterwards, samples were centrifuged for 15 min at 2000× *g* at 4 °C, then aliquoted and stored at −80 °C until further analysis. Subsequently, mice were sacrificed with sodium pentobarbital (250 mg/kg, i.p.).

Cases of sudden death during the experiment were documented. All animal procedures were approved by the ethics committee of the University of Antwerp (EC nr. 2014-15; 1 April 2014) and complied with the guidelines from Directive 2010/63/EU of the European Parliament on the protection of animals used for scientific purposes.

### 2.2. Activity Assay for the Measurement of ProCPU in Plasma of Mice

To accurately determine the proCPU concentration in mouse plasma samples, preanalytical and analytical variables (including the thrombin–thrombomodulin concentration, preincubation time, preincubation temperature and substrate concentration) of an in-house, activity-based human proCPU assay were optimized. EDTA plasma from female ApoE^−/−^ mice on a normal diet (N = 6) and the following reagents were used for the optimalization: rabbit-lung thrombomodulin (Seikisui Diagnostics, Burlington, Massachusetts, USA), Bz-*o*-cyano-Phe-Arg (Laboratory of Medicinal Chemistry, University of Antwerp, Belgium), 4-(2-hydroxyethyl)-1-piperazineethanesulfonic acid (HEPES), human thrombin, D-phenylalanyl-L-prolyl-L-arginine chloromethyl ketone (PPACK) and CaCl_2_ (all from Merck, Darmstadt, Germany) [11]. The within and between-run imprecision of the adapted assay was determined, and the cut-off oxyhemoglobin (oxyHb) level—resulting in a 10% reduction of the proCPU concentration—was defined according to Mertens et al. using hemolysate obtained by the lysis of red blood cells from ApoE^−/−^ mice on a normal diet (N = 6) [12].

Subsequently, plasma proCPU levels of ApoE^−/−^Fbn1^C1039G+/−^ mice on WD and WD + atorvastatin therapy were measured with the newly validated assay. The method described by Kahn et al. was used to determine cell-free oxyHb levels in all samples [13].

### 2.3. Measurement of Total Plasma Cholesterol, CRP and Blood Immune Cells

Total plasma cholesterol and C-reactive protein (CRP) were measured with commercially available ELISA kits (Total cholesterol, Randox, Crumlin, UK and MCRP00, R&D systems, Minneapolis, MN, USA respectively).

Red blood cells in EDTA whole blood were lysed (red blood cell lysing buffer Hybri-Max, Sigma, St. Louis, MO, USA), followed by the labeling of the remaining leukocytes (monocytes, neutrophils and dendritic, natural killer (NK), natural killer T (NKT) and T-cells). Next, labeled leukocytes were analyzed by flow cytometry as described previously [10].

### 2.4. Statistical Analysis

All data were expressed as mean ± standard deviation (SD). Statistical analysis and data plotting were performed using GraphPad Prism version 9 (GraphPad Software, Inc. La Jolla, San Diego, CA, USA). Statistical tests are specified in the figure legends. Results were considered significant at *p* < 0.05.

## 3. Results and Discussion

### 3.1. Activity Assay for the Measurement of ProCPU in Mice

**Figure 1:**
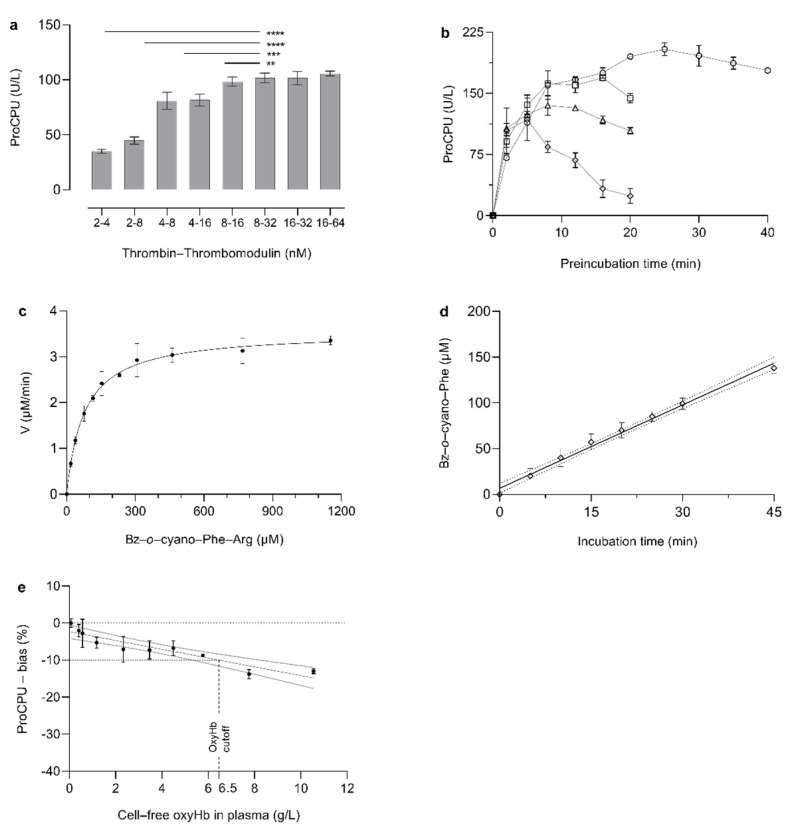
(**a**). Optimization of thrombin and thrombomodulin concentration. Plasma samples were activated with 50 mM CaCl_2_ and different concentrations of human thrombin and rabbit-lung thrombomodulin in different ratios. Samples were diluted 10-fold in 20 mM HEPES prior to activation. After 10 min of preincubation with the thrombin–thrombomodulin mixture at 25 °C, CPU activity was quantified. Data represent mean ± SD (N = 3). A Kruskal-Wallis test with Dunn’s multiple comparison test was used to test for statistical significance between all groups. The significance levels of **, ***, **** correspond to *p*-values of <0.01, <0.001 and <0.0001, respectively. (**b**). Effect of preincubation temperature and time interval on proCPU activation. Plasma samples were diluted 10-fold in 20 mM HEPES (4-(2-hydroxyethyl)-1-piperazineethanesulfonic acid). Preincubation with 8 nM human thrombin and 16 nM rabbit-lung thrombomodulin was performed at 10 °C (dots), 15 °C (squares), 20 °C (triangles) and 25 °C (diamonds). At different time points, the reaction was stopped with PPACK (D-Phenylalanyl-L-prolyl-L-arginine chloromethyl ketone). CPU activity was determined by adding Bz-*o*-cyano-Phe-Arg (900 μM), and the formation of Bz-*o*-cyano-Phe was measured by reversed phase high-performance liquid chromatography (RP-HPLC). Data are presented as mean ± SD (N = 2). (**c**). Michaelis–Menten curve of Bz-*o*-cyano-Phe-Arg cleavage by CPU in mouse plasma. ProCPU in pooled mouse plasma was quantitatively activated. The activation was stopped with PPACK followed by incubation with different concentrations of Bz-*o*-cyano-Phe-Arg (0–1150 µM). The initial velocities of product formation were plotted against the different substrate concentrations. A Km value of 89 ± 4 μM was obtained. Data are presented as mean ± SD (N = 4). (**d**). Linearity of substrate conversion. ProCPU in pooled mouse plasma was activated with thrombin–thrombomodulin followed by incubation with 900 μM of Bz-*o*-cyano-Phe-Arg at 25 °C for different periods of time. The formation of the reaction product Bz-*o*-cyano-Phe was plotted against the incubation time. Data represent mean ± SD, N = 2. The linear regression curve and its 95% CI (dotted lines) are displayed in black. Linear substrate conversion was achieved up to 45 min. (**e**). Influence of hemolysis on proCPU levels. Different concentrations of oxyhemoglobin (oxyHb; final concentration 0–10.5 g/L) (hemolysate) were spiked in mouse plasma and proCPU levels were measured. The bias in the proCPU concentration—compared to the nonhemolytic reference level (black dotted line)—was plotted for each oxyHb concentration. Data are presented as mean ± SD (N = 3). The linear regression curve (black line) and its 95% CI (grey lines) are displayed. The cut-off oxyHb level (black dashed lines)—resulting in a 10% reduction of the baseline proCPU concentration—was 6.5 g/L.

Our in-house assay for proCPU measurement in human citrated plasma was adapted for use in mice EDTA plasma [11]. The quantitative activation of proCPU in mice samples was found to be optimal when plasma was diluted 10 times in HEPES (20 mmol/L, pH 7.4) followed by incubation with 8 nM purified human thrombin ), 16 nM rabbit-lung thrombomodulin and 50 mM CaCl_2_ for 25 min at 10 °C (Figure 1a,b).

To determine proCPU activity, the generated active CPU was incubated for 15 min at 25 °C with the specific and selective substrate Bz-*o*-cyano-Phe-Arg (900 µM final concentration) (Figure 1c,d). Subsequently, the formed product was quantified by high-performance liquid chromatography as previously described [11]. For this assay, 1 unit of enzyme activity was defined as the amount of enzyme required to hydrolyze 1 µmol of substrate per minute at 25 °C under the conditions described.

The modified assay proved to be precise (within-run CV = 2.3%, between-run CV = 4.9%) and oxyHb levels up to 6.5 g/L are allowable (Figure 1e).

### 3.2. Total Plasma Cholesterol

**Figure 2:**
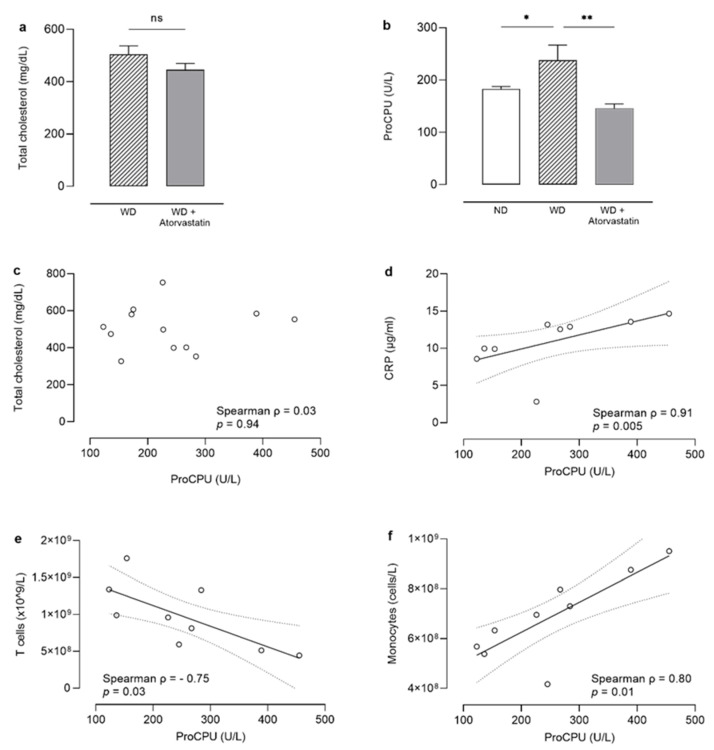
(**a**). Bar graph showing total plasma cholesterol levels in ApoE^−/−^Fbn1^C1039G+/−^ mice on a Western diet (WD) (N = 12) or WD supplemented with atorvastatin (WD+Atorvastatin) (N = 17). Data presented as mean ± SD. A Mann–Whitney U test was used to test for statistical significance between both groups; *p* > 0.05. (**b**). Bar graph showing plasma proCPU levels in the WD group (N = 12), WD + Atorvastatin group (N = 15) and in ApoE^−/−^ mice on a normal diet (N = 6). Data presented as mean ± SD. A Mann–Whitney U test was used to test for statistical significance; * *p* < 0.05, ** *p* < 0.01. (**c**–**f**). Relation between plasma proCPU levels and total plasma cholesterol (**c**), C-reactive protein (CRP) (**d**), circulating T cells (**e**), circulating monocytes (**f**). Spearman correlation coefficient ρ was determined for all correlations. In case of a significant correlation (*p* > 0.05), linear regression analysis was performed, and the best-fit line (solid line) with 95% confidence bands was plotted (dashed lines).

Total cholesterol (TC) was measured in plasma of ApoE^−/−^Fbn1^C1039G+/−^ mice that were fed a WD (N = 12) or a WD combined with atorvastatin (WD+atorvastatin; 10 mg/kg/day; N = 17). Results confirmed that atorvastatin did not significantly reduce TC in these mice (*p* = 0.19; Figure 2a).

### 3.3. ProCPU Decrease in Atorvastatin-Treated Mice on a Western-Diet Is Cholesterol-Independent

After adapting our in-house activity-based proCPU assay for use in mouse EDTA plasma, plasma proCPU levels were determined, and the effect of atorvastatin on proCPU biology in a cholesterol-independent setting was evaluated. ProCPU levels were found to be significantly lower in mice receiving WD + atorvastatin compared to mice on control WD (159 ± 63 U/L [range 97–227 U/L] vs. 238 ± 101 U/L [range 123–455 U/L]; *p* = 0.004; Figure 2b). Similar to observations made in humans, atorvastatin reduced proCPU levels in ApoE^−/−^Fbn1^C1039G+/−^ mice fed a WD, resulting in plasma proCPU concentrations similar to those found in ApoE^−/−^ mice on a normal diet (178 ± 32 U/L [range 165–210 U/L]; *p* = 0.24; N = 6) [8]. The study design did not allow us to determine the change in proCPU levels before and after treatment with atorvastatin in individual mice. Therefore, it was not possible to establish whether the largest proCPU decrease is seen in mice with the highest baseline proCPU levels—something that was perceived in humans receiving statin therapy [8].

In addition, no correlation was observed between plasma proCPU and TC in the WD group (Figure 2c). In this model, the downregulation of proCPU levels is thus a cholesterol-independent, pleiotropic effect of atorvastatin treatment. A possible mechanism for this downregulation is related to peroxisome proliferator-activated receptor α (PPARα). PPARα participates in the regulation of various aspects of lipid metabolism in the liver, finally resulting in hypolipidemic effects [14,15]. Kilicarslan et al. described that fenofibrate, a PPARα agonist, decreased proCPU levels in patients with metabolic syndrome, suggesting that agonists of PPARα possess anti-thrombotic properties through the decrease of circulating proCPU levels on top of their role as anti-lipidemic agents [16]. Moreover, Masuda et al. reported on the downregulation of the *CPB2* gene expression in HepG2 cells and decreases in both CPB2 mRNA and proCPU antigen levels, mediated by the PPARα signaling pathway upon treatment with the PPARα agonist WY14643 [17]. Since it has been described that statins increase PPARα expression (although they are not direct ligands for PPARα), the hypothesis that statin therapy could increase PPARα expression—which in turn could lead to reduced *CPB2* gene expression and thus lower plasma proCPU levels—seems plausible but was not further explored in the current work [6,18,19,20].

### 3.4. Inflammation and Blood Immune Cells

Alongside TC and proCPU levels, plasma CRP and circulating blood immune cells were also measured, revealing that atorvastatin significantly improved the inflammatory blood profile in ApoE^−/−^Fbn1^C1039G+/−^ mice with significant reductions in plasma CRP (*p* = 0.001) and circulating monocytes (*p* < 0.01; Table 1). T cells were significantly increased (*p* = 0.01; Table 1). Possible correlations between these parameters and proCPU concentrations were investigated in the WD-fed mice. A clear positive association was found between plasma proCPU and CRP (ρ = 0.91, *p* = 0.005; Figure 2d), while a negative association was observed between circulating T cells and plasma proCPU (ρ = −0.75, *p* = 0.03; Figure 2e). Moreover, proCPU levels also correlated with circulating monocytes (ρ = 0.80, *p* = 0.01), meaning that higher proCPU levels occur together with high amounts of these immune cells (Figure 2f). Besides liver-derived proCPU, the existence of extra-hepatic proCPU in megakaryocytic and monocytic cell lines (cell lysates and conditioned media) has been reported [21,22]. Lin and co-workers also suggested that these cells may be a source of proCPU within atherosclerotic plaques as well as in other extra-vascular sites during inflammation [21,23]. Hence, monocyte-derived proCPU could contribute to the increase in plasma proCPU concentration in WD mice, although presumably only to a limited extent since the liver remains the main source of circulation proCPU [24]. Interestingly, it was demonstrated that inflammatory cytokines (both pro and anti-inflammatory), which are abundantly present and upregulated in atherosclerosis, increase proCPU secretion in monocytes [21,25]. Consequently, the reduction of inflammatory cytokines and circulating monocytes induced by atorvastatin therapy could (partly) be attributed to the decrease in proCPU levels that is also seen with this therapy.

The importance of the correlation between proCPU and circulating T cells is not clear; in particular, because—to the best of our knowledge—*CPB2* expression and regulation have not yet been studied in T cells, complicating the correct interpretation of these results.

## 4. Conclusions

In conclusion, our results confirm that atorvastatin downregulates proCPU levels in ApoE^−/−^Fbn1^C1039G+/−^ mice on a WD, in line with the observations in humans. As a result, this therapy improves the fibrinolytic capacity in addition to its lipid-lowering properties. Evidence is provided that this downregulation is a pleiotropic effect of statin treatment. Furthermore, this report is, to our knowledge, the first to describe a correlation between plasma proCPU levels and certain circulating immune cells. Elucidating the role of these immune cells in the atorvastatin-induced downregulation of the CPU system and looking into the involvement of the PPARα pathway will provide valuable information to help unravel the molecular mechanism by which atorvastatin modulates plasma proCPU levels.

## Figures and Tables

**Table 1:** Inflammation and blood immune cells.

Parameter	WD	WD + Atorvastatin	*p*-Value
CRP (µg/mL)	10.9 (3.6)	7.4 (2.6)	0.001
Leukocytes (10^9^/L)	6.02 (2.42)	6.77 (2.10)	0.35
Monocytes (10^9^/L)	0.62 (0.18)	0.37 (0.26)	0.01
Neutrophils (10^9^/L)	1.54 (1.11)	0.78 (0.55)	0.08
Dendritic cells (10^9^/L)	0.062 (0.057)	0.087 (0.035)	0.16
T cells (10^9^/L)	0.88 (0.24)	1.49 (0.33)	0.01
NK cells (10^9^/L)	0.51 (0.22)	0.49 (0.17)	0.93
NKT cells (10^9^/L)	0.063 (0.041)	0.066 (0.028)	0.49

Abbreviations: CRP: c-reactive protein; NK cells: natural killer cells; NKT cells: natural killer T cells; WD: ApoE^−/−^ Fbn1^C1039G+/−^ on a western diet (N = 9–12); WD + atorvastatin: ApoE^−/−^ Fbn1^C1039G+/−^ receiving a Western diet supplemented with atorvastatin (10 mg/kg/day) (N = 15–17). Note: Data are expressed as mean ± standard deviation.

## Data Availability

The data presented in this study are available on request from the corresponding author.
